# Letter to the editor regarding Gholamzad et al., “A comprehensive review on the treatment approaches of multiple sclerosis: currently and in the future”

**DOI:** 10.1007/s00011-019-01310-3

**Published:** 2020-01-11

**Authors:** Simon Faissner

**Affiliations:** Department of Neurology, St. Josef-Hospital, Ruhr-University Bochum, Gudrunstr. 56, 44791 Bochum, Germany

Dear Sirs,

In their review entitled “A comprehensive review on the treatment approaches of multiple sclerosis: currently and in the future”, Gholamzad et al. also write about the CD25 targeted injectable antibody daclizumab [1]. While the authors correctly describe its mechanism of action and trial data as well as the initial approval status, they, unfortunately, do not state that daclizumab has been retracted from the market in March 2018 due to 12 cases of serious inflammatory brain disorders, including encephalitis and meningoencephalitis with three fatal cases [2]. The mechanism of secondary autoimmunity is until now not entirely understood. One explanation for the development of daclizumab-associated secondary autoimmunity might be the fact that the expansion of regulatory T cells and possibly activation induced T-cell apoptosis might be impaired [3]. Therefore, post-marketing vigilance is crucial to ensure safety in patients treated with new medications [4] and learn about new and as of unknown side effects. Since the review appeared online half a year after the retraction of daclizumab from the market, this information certainly should have been included.
